# Suicide Risk Assessments: A Scientific and Ethical Critique

**DOI:** 10.1007/s11673-022-10189-5

**Published:** 2022-05-23

**Authors:** Mike Smith

**Affiliations:** Bioethics Centre, University of Otago, Dunedin, New Zealand

**Keywords:** Suicide, Risk assessment, Ethics, Screening, Prevention, Paternalism

## Abstract

There are widely held premises that suicide is almost exclusively the result of mental illness and there is “*strong evidence for successfully detecting and managing suicidality in healthcare”* (Hogan and Grumet, 2016). In this context, ‘zero-suicide’ policies have emerged, and *suicide risk assessment* tools have become a normative component of psychiatric practice. This essay discusses how suicide evolved from a moral to a medical problem and how, in an effort to reduce suicide, a paternalistic healthcare response emerged to predict those at high risk. The evidence for the premises is critiqued and shown to be problematic; and it is found that s*trong* paternalistic interventions are being used more often than acknowledged. Using a Principles approach, the ethics of overriding autonomy in suicide prevention is considered. Ethical concerns are identified with the current approach which are potentially amplified by the use of these risk assessments. Furthermore, it is identified that the widespread use of risk assessments in health settings is equivalent to screening without regard to the ethical principles of screening. The essay concludes that this is unethical; that we should abandon the use of standardized suicide risk assessments and ‘zero-suicide’ policy; and that this may improve outcomes.

*And the more pity that great folk should have count’nance in this world to drown or hang themselves more than their even-Christian.*

- William Shakespeare. *Hamlet* (act 5, scene 1, lines 25–28) (Shakespeare 1987)

## Introduction

The World Health Organization (2018) states that there is a global imperative to address suicide rates, and describes suicide, which results in an estimated 800,000 deaths per year worldwide, as preventable. Historically, the elderly have been affected by suicide in the highest numbers, and globally this continues to be the case. There has been a trend down the age groups, however, and suicides among young people now account for nearly a third of the total (Hoven et al. 2010). There are significant variations in suicide rates between countries and across cultures (Ritchie, Boser, and Ortiz-Ospina 2015). The last twenty years have seen increases in suicide rates in Australia (Australian Institute of Health and Welfare 2021), whereas New Zealand has relatively stable rates (Coronial Services of New Zealand 2020) but is in the unenviable position of having the highest rate of suicide among youth in the world, with young male Māori significantly more likely to die by suicide than their non-Māori counterparts (Coronial Services of New Zealand 2021).

Recent international responses to suicide rates have been comparable, with strategies to reduce rates that include increasing awareness and better understanding, improving access to mental health care, and reducing access to lethal means (United States Surgeon General and National Action Alliance for Suicide Prevention 2012; New Zealand Ministry of Health 2019; NHS England 2016).

Within the last decade the policy of “zero suicide” has emerged. First proposed by the U.S. Surgeon General’s Clinical Care and Intervention Taskforce (United States Surgeon General and National Action Alliance for Suicide Prevention 2012), the basis of “zero suicide” is “strong evidence for successfully detecting and managing suicidality in healthcare” (Hogan and Grumet 2016, 1086). The idea is, if this can be done effectively enough, suicide rates could approach zero.

In this context a major area of focus in suicide prevention initiatives has been the link between suicide and mental illness. There is a widely held premise that suicide is almost exclusively the result of mental illness. A logical conclusion is that by detecting people who are at high risk of suicide in health settings, and treating their mental illness, suicide rates will decline. Significant research efforts have gone into suicide risk assessment tools, and zero suicide policy has been adopted by many healthcare providers and at least one national government.^1^ As a component of these efforts, there has been a strong emphasis on risk assessment becoming a normative part of psychiatric practice, and local health authorities have supported this with the roll-out of ongoing suicide risk training to clinicians, to ensure standardization of this process. Providing momentum to all of this is the widespread appeal, to clinicians, hospital managers, families, and patients, of the idea that suicide can be predicted, and risk of later suicide quantified (Mulder, Newton-Howes, and Coid 2016).

As a psychiatrist who works within the public health system I have observed, at first hand, the implementation of this policy. In this essay I will discuss the history and the scientific arguments behind the emergence of the policy, beginning with consideration of how suicide evolved from a moral and criminal issue to become conceptualized as a medical problem that occurs primarily as a result of mental illness and how, in an effort to reduce suicide rates, a paternalistic healthcare response emerged to predict those at high risk of suicide through the widespread use of “suicide risk assessments” as part of “zero-suicide” policy in mental healthcare. I will then examine the policy, critiquing contemporary evidence and in doing so identify that a strong paternalistic intervention, overriding autonomy, is being used more often than is being acknowledged. Using a principlist approach, I consider the ethical justification for overriding autonomy in suicide prevention and find significant issues relating to beneficence, non-maleficence, and justice from the use of suicide risk assessments in individuals. Furthermore, I identify that the widespread use of risk assessments is consistent with a screening programme, but that recognized ethical principles of screening have not been applied, and that this approach is unethical. To conclude I will discuss how restricting liberty in an effort to prevent suicide is likely to continue, but that abandoning the use of suicide risk assessments and zero-suicide policy in healthcare may reduce the use of coercive interventions and encourage a shift in focus and resources towards improved access to mental health treatment and towards strategies that do have evidence for improving suicide prevention efforts.

## Suicide: From Morality to Medicalization

Suicide has been a focus of ethical debate from antiquity. Aristotle proposed that suicide to escape from poverty, pain, or sorrow was cowardly and a failure of courage (Aristotle 1926). Major deontological theories, both religious and secular, also viewed suicide as morally wrong. Kant believed that human life had intrinsic worth and the act of suicide would be to use oneself as a means (to end suffering) rather than an end, writing in *Groundwork of the Metaphysics of Morals* (Kant 2002) that to “preserve one’s life is a duty.” Consequentialists have held more diverse positions on suicide, identifying that each situation is unique but that suicide is not inherently immoral and autonomous people have a right to self-determination (Mishara and Weisstub 2005).

Reflecting these different moral views, a range of beliefs exists about the nature of any intervention to prevent suicide: from the moralist position that suicide is wrong and there is an obligation to protect life to consequentialist positions that individual circumstances determine the nature of intervention. John Stuart Mill, the prominent utilitarian thinker, did not directly reference suicide but did suggest that liberty could be temporarily suspended to check the person knew what the consequences of their intended actions would be (Mill 2003). Despite a variety of beliefs, there is generally (strong libertarians aside) a shared understanding within society that some form of intervention in someone who is suicidal is appropriate. A foundation of this shared belief is the longstanding idea of a social contract, which gained increasing authority through the ideas of political philosophers such as John Locke. In *Treatises of Government*, Locke outlined a social contract in which free and equal people transfer some of their rights to the government in order to better ensure the stable, comfortable enjoyment of their lives, liberty, and property (Locke 1995).

Historically, powers of social control have sat with the state and religion, but over time this has shifted. *Medicalization* is the process by which human problems enter the domain of the medical profession. A problem comes to be defined in medical terms, a medical framework is used to understand it, and/or a medical intervention used to “treat it”. A number of reasons have been posited as to why medicalization can occur, for example, increased secularization with the diminution of religion; faith in science, rationality, and progress; and increased prestige and power of the medical profession (Conrad 1992). One of the consequences of medicalization, however, is the transfer of some powers of social control from the state to the medical profession (Zola 1972; Conrad 1979). A common assumption with such a transfer is that the social problem at hand is removed from religious and legal scrutiny and their associated moral and punitive consequence and placed instead under medical and scientific scrutiny which will result in (more benign) objective and therapeutic circumstances (Zola 1972).

While a full examination of the process of medicalization is beyond the scope of this essay, in the case of suicide it has been suggested that as a result of secularization, and associated “loss of diabolical powers,” suicide became medicalized almost by default (MacDonald 1989; Conrad 1992). If this was the case, the medicalization process was also considerably eased by the increasing acceptance of an association between suicide and mental illness and the emergence of the *medical model* of suicide.

In considering the origins of the medical model, it is worth noting that as monotheistic religions emerged, and their doctrine evolved, the view that suicide was wrong increasingly took hold. From Augustine onwards, the Christian Church condemned suicide as a sin (Battin 2005). When the Church’s influence spread, this was reflected in societies, where suicide was frequently criminalized. In Britain, suicide became subject to harsh punishment under common law and Christian doctrine from the thirteenth century (MacDonald 1989). To be found guilty of attempted “self-murder” risked forfeiture of property and, ironically, the death penalty.

This view of suicide as sin and requiring punishment was not held ubiquitously. As far back as the sixteenth century, the physician Robert Burton, in his work *The Anatomy of Melancholy* (Burton 1932), considered suicide to be the outcome of melancholy. On this background, mental illness was increasingly linked to suicide by juries. Accused were increasingly found *non compos mentis* (not of sound mind) rather than *felo de se* (a felon of himself), as a way for juries to avoid imposing punishments viewed as harsh even for the time.

Despite early insights such as Burton’s, in medical circles the association between suicide and mental illness remained one of considerable debate. Many prominent physicians did recognize some form of association. *Melancholia* continued to be emphasized by a number of authors in the nineteenth century,^2^ but this was often accompanied by a note of caution, with a reference to the relevance of social factors and the apparent lack of mental illness in some cases of suicide (Goldney and Schioldann 2000, Goldney, 2003). In their influential textbook *Manual of Psychological Medicine*, British physicians Bucknill and Tuke cautioned that “it cannot be admitted for a moment that the suicidal act taken alone is any sign of insanity” (Bucknill and Tuke 1858). Many physicians continued to retain deep scepticism about the link between mental illness and suicide, noting the suicide often occurred in the absence of pathology and rather as a result of recognized psychosocial stressors. As late as 1982, Hawton and Catalan (1982) noted that only a minority of patients who have attempted suicide suffer from formal psychiatric illness requiring psychiatric treatment (Goldney 2003).

Sociopolitical changes continued to influence societal views of suicide rather than medical science (Houston 2009). From the Age of Enlightenment, and through the eighteenth and nineteenth centuries, Western societies progressed towards more relative freedom of thought and the pursuit of knowledge and understanding. Emerging liberal attitudes, influenced by the work of theorists such as Durkheim and Freud, became a force for decriminalization of suicide. Concurrently, the power of the church was gradually diminishing.

Then, with the emergence of suicide research in the latter third of the twentieth century, scientific weight was added to public opinion. Harris and Barraclough (1997), for instance, published a meta-analysis of the association between suicide and various mental and physical disorders and showed, among other results, rates of suicide thirty-five times higher than expected in people experiencing depression. At around the same time, a series of psychological autopsy studies concluded that mental illness was present in ninety per cent of people who completed suicide (Cavanagh et al. 2003). The statistic has since become a well-known, often quoted, “truth,” and the medical model has taken a dominant place in suicide discourse.

## Bioethics and Suicide Prevention

In liberal democracies, where legal recourse is available against unlawful or arbitrary detention by the state by way of a writ of *habeas corpus,* there is the potential for uncertainty in how to legally intervene to limit the liberty of someone who is suicidal and what the nature of the intervention should be. If suicide is the result of mental illness, then these uncertainties, both ethical and legal, are made redundant. The medical model provides a straightforward pathway for addressing suicide through existing healthcare systems. In many jurisdictions, a legal framework exists outside of the criminal justice system—mental health legislation—to detain someone who is suicidal for further assessment on the grounds that they may be experiencing mental illness. An important societal ethical question is replaced with medical ethics of paternalism, relating to treatment of the mentally unwell.

Emerging with the Nuremberg Code (1947) (Nuremberg Military Tribunal 1996) and underlined by the Belmont Report (1978) (Department of Health, Education and Welfare; National Commission for the Protection of Human Subjects of Biomedical and Behavioral Research, 2014), the importance of patient rights and, specifically, informed consent and the underlying principle of respect for autonomy has become firmly embedded in biomedical ethics and health law. Against this background, and with recognition that this principle requires balancing against other, potentially conflicting, principles, Beauchamp and Childress’s landmark work, *Principles of Biomedical Ethics* (Beauchamp and Childress 2019), has established itself as a respected and widely used framework for considering ethical issues in healthcare.

The problem of whether, or the extent to which, the autonomy model is applicable to patients experiencing mental illness has been considered by Radden (2002) in her work on psychiatric ethics. Radden outlines the problem with viewing the patient as an autonomous agent when, by virtue of mental illness, they may be deprived of the capabilities, temporarily or partially, required for the exercise of autonomy. A central tension exists. There is an exaggerated degree to which mentally ill people are considered to be non-autonomous, perhaps perpetuating from a stigmatizing attitude towards people with mental illness, when many people continue to function and retain their capacity for decision-making despite experiencing mental illness. It is equally important, however, not to misapply the autonomy model and attribute it where it is compromised. Doing so risks the creation of injustice and could cause harm. Radden concludes that the principles are applicable and necessary but not sufficient in considering bioethical issues in psychiatry and proposes that the recognized, and common, approach to addressing this dilemma is the ongoing use of paternalism towards psychiatric patients, albeit a form that is “compassionate” or “weak.”

Although a universally accepted definition of paternalism has evaded bioethicists (Dworkin 2015), Radden refers to a preference-limiting definition, where the paternalistic action can be directed towards limiting both autonomous and non-autonomous choices (Beauchamp 2014). This definition is felt by many to be truer to the origins of the term, taking into account its role in the protection of the vulnerable, and is a definition I favour in this essay. Feinberg (1971) first differentiated between autonomous and non-autonomous paternalism, using the terms “strong” and “weak” respectively. This is an important distinction when considering paternalism using a principlism approach. With no set lexical order of principles, balancing autonomy against beneficence (and non-maleficence) is an important task.

In mental health settings, and specifically suicide prevention efforts in mental healthcare, there is a presumption that the paternalism being used is weak. If someone wants to kill themselves, they are mentally ill and non-autonomous. Preventing this harmful act allows that person to go on and receive effective treatment for their mental illness, the restoration of their autonomy, and their return to a non-suicidal condition.

## A Critique of the Evidence and Ethics of Suicide Risk Assessments

A key component of suicide prevention in healthcare, at the level of the clinician–patient interaction, has been the widespread emergence of a policy of using systematized suicide risk assessments in mental health settings. The idea is that these assessments are conducted on everyone who is seen by mental health services and are a normative part of ongoing health interactions. Studies showing that significant numbers of people who died by suicide were in contact with their primary healthcare provider shortly before they died are positioned as evidence for an opportunity to detect those at risk; for example, a systematic review by Stene-Larsen and Reneflot (2019) of forty-four studies found that on average forty-four per cent were in contact with primary care in the month before they died, and eighty per cent in the preceding year. The premises for this policy are that by identifying these people through widespread use of risk assessments and treating their underlying mental illness, coercively if necessary, suicides will be successfully prevented.

There are concerns, however, that evidence which has been used to argue in favour of such a strong correlation between suicide and mental illness, and which underlies the medical model, is not as robust as has been accepted. Methodological flaws in psychological autopsy studies have been identified by several groups (Hjelmeland et al. 2012; Pouliot and de Leo 2006). A psychological autopsy involves interviewing someone who knew the deceased to determine if they had a mental illness. Two fundamental problems arise from this approach. It is impossible to assign a reliable diagnosis on a person by interviewing someone else. Then there are problems with recall bias from a grieving relative attempting to make sense of the suicide or preconceived beliefs that mental illness must be the explanation. Problematic also is that the very high rates of mental illness found in the earlier studies have not been replicated in more contemporary studies. Subsequent studies have observed rates closer to sixty per cent (Hirokawa et al. 2012; de Leo et al. 2013; Goldney 2003) and found that multiple risk factors are present when a suicide occurs, with psychiatric diagnosis not a significant predictor of suicide (Phillips et al. 2002). Some authors note that rates of suicide appear to be unrelated to the prevalence of mental illness (e.g., Pouliot and de Leo 2006).

Other disciplines also cast doubt about such a strong causal relationship between mental illness and suicide. The nineteenth century sociologist Emile Durkheim (2002), in an extensive study, concluded that suicide was inversely correlated to social integration. He identified risk factors still recognized as important today: loss of employment, financial losses, family troubles, suicides of criminals, physical sufferings, drunkenness, and mental illness.

Contemporary studies have gone a long way to validate Durkheim’s findings. Blakely, Collings, and Atkinson (2003) observed that unemployment resulted in a threefold risk of suicide, of which only half was explainable by mental illness. Epidemiological studies have confirmed social isolation as being a significant factor, (Agerbo, Stack, and Petersen 2011; Qin, Agerbo, and Mortensen 2003). Higher rates among prisoners have been identified in several studies. One large national study observed that one-third of prison related suicides occurred within the first week, and of these under sixty per cent had been identified as having a mental illness a few days before (at reception), with depression noted in very few (Shaw et al. 2004). Savage (1892) and Durkheim (2002) also described suicides that were “altruistic,” rather than the result of illness or despair. These occurred more often in excessively integrated societies and are perhaps best recognized from Japan, where they are called *kakugo no jisatsu* (suicide of resolve) and considered a rational act (Kitanaka 2008).

In many cases it will be the case that mental illness is present and is a contributing factor. Findings such as those outlined here pose a major challenge to the presumption that suicide is overwhelmingly the result of mental illness, however, and indicate a need to move away from a simplistic view that ninety per cent of suicides occur as a result of depression or mental illness.

This leads us to consider the current use of suicide risk assessment, with subsequent interventions for people stratified as high risk justified by weak paternalism. Founded on an argument that suicidal people are mentally ill and rendered non-autonomous by their illness, this is relatively uncontroversial. The highlighting that suicide is complex and not simply the consequence of mental illness does not in itself change this argument when mental illness remains a factor. The absence of mental illness, however, in a significant minority of people who suicide and the existence of rational suicide suggests the likelihood that a significant minority retain their autonomy. Strong paternalism is therefore being used more often than is implied in healthcare-based suicide prevention efforts.

When viewed from a principlist perspective, where respect for autonomy is in balance with beneficence and non-maleficence, this shifts the balance. Weak paternalism is more likely considered benign, and uncontroversial, having a significant degree of acceptance and acceptability within society. Strong paternalism can be justified, but there is a burden to provide adequate reasons for the action (Childress 2020). Beauchamp and Childress (2019) state the conditions which must be satisfied for strong paternalism:

…risk of a significant, preventable harm or failure to receive a benefit, that the paternalistic action will probably prevent; the intervention outweighs the risks to the patient of the action taken; there is no morally better alternative; and the least autonomy-restrictive alternative that will prevent the harm or secure the benefit is adopted. (Beauchamp and Childress 2019, 238)

In considering the evidence to justify overriding autonomy through strong paternalism after stratifying someone as high risk by a risk assessment, it is necessary to examine the validity of the strategies that are critical for predicting who is going to end their life by suicide, and the evidence for suicide interventions, to confirm they provide support for beneficence and non-maleficence, rather than harm.

### Evidence for Prevention

The widespread use of suicide risk assessments to reduce suicide rates will, by the policy’s design, result in increased prediction of suicide risk and increased intervention, both coercive and non-coercive. The risks of a paternalistic coercive, or compulsory, intervention in mental health care have been documented. As noted earlier, while the transfer of powers of social control to manage suicide risk over to medical settings can be intended, or perceived, to be a move away from negative moral or punitive consequences and toward therapeutic consequences, in reality coercive psychiatric intervention is overwhelmingly seen as negative by patients (Newton-Howes and Mullen 2011), is associated with future unwillingness to disclose suicidal feelings or intentions (Jones et al. 2021), and has been shown to potentially increase the risk of suicide (Jordan and McNiel, 2020; Large et al. 2014). There are also significant imbalances between rates of compulsory intervention across cultures. As noted earlier, suicide disproportionately affects young Māori males in New Zealand. It is also the case that this same group is overrepresented in terms of being subject to compulsory assessment and treatment under the Mental Health Act (New Zealand Ministry of Health 2020a). A similar pattern is seen in other countries, for example with indigenous rates of suicide in Canada (White and Morris 2019). Any increase in compulsion is likely to disproportionately affect these already overrepresented groups, compounding injustice, and appearing to conflict with the principle of non-maleficence.

There is a further concern when considering potential health improvements from any intervention, whether coercive or non-coercive: even if the predictive powers of the assessment tools were greatly improved, the evidence for specific individual interventions for suicide reduction is alarmingly small. At the time of writing, I have been unable to identify a single evidence-based suicide intervention for an individual. There is evidence for specific interventions for specific mental disorders, e.g., lithium for bipolar disorder (Cipriani et al. 2005), but even here reduction, not elimination, is observed. This is insufficient for the objective of “zero suicide” and does not provide much support demonstrating beneficence to justify a strong paternalistic action.

### Evidence for Prediction

Structured risk assessments and clinical evaluation are the strategies that have been developed for detecting and predicting suicide. Large et al. (2017) identified six high quality meta-analyses of suicide risk assessments, none of which found any single risk factor or combination of risk factors associated with later death by suicide. Two of the meta-analyses found that suicide risk assessments had a positive predictive value (PPV) 3 of five per cent in the long term (Carter et al. 2017; Large et al. 2016). Studies of “structured clinical evaluation” found that they were not superior to predictive instruments. When professionals overrode statistical predictions, validity decreased (Garb and Wood 2019). The commonality of suicide risk assessment strategies appears to be their lack of utility at meaningfully predicting risk (Franklin et al. 2017; Runeson et al. 2017).

### Normative Suicide Risk Assessment and the Ethics of Screening

Critics may argue that any issue of coercion will apply to only a small proportion of people assessed, so harm is limited. This is unknown, but there is a wider ethical concern affecting all participants. Suicide risk assessment, as a method of identifying or predicting those at increased risk of suicide, is a form of screening. This screening is usually targeted at patients in contact with mental health services; however, an extension into primary care has been proposed (Finnegan, Selwyn, and Langhinrichsen-Rohling 2018; Raue, Ghesquiere, and Bruce 2014). Ethical considerations for any screening programme, targeted or otherwise, are widely recognized, and include informed choice, confidentiality, respect for autonomy, and the balancing of benefits and harm (Andermann et al. 2008).

There is an acceptance that a person to whom screening is directed should know the facts about the screening test on offer so as to be able to decide whether to have that test (Delatycki 2012). This is the concept of informed choice, an essential component of protecting autonomy. With suicide risk assessments now a normative component of mental health interactions there is a valid question of when, or if, patients are even aware that they are being screened, let alone given the opportunity to enact a choice. That this is the case is a significant breach of the principle of autonomy and the right of people to choose, free from coercion, whether they want to participate. It is recognized that obtaining truly informed consent for all screening is probably not possible. This would require the provision of detailed information about the screening test and the person to whom the test is directed taking the time (or having the ability) to understand it—this is overdemanding (Beauchamp and Faden 2014). Given, however, that the majority of mental health care interactions are not the result of a person making a suicidal gesture, when suicide risk assessments are being used normatively in these interactions, there appear to be no reasonable ethical justification not to uphold generally accepted elements of gaining informed consent for these assessments, which are (1) disclosure, (2) understanding, (3) voluntariness, (4) competence, and (5) consent (see Beauchamp and Faden 2014). In New Zealand these are also legal rights that are enshrined in the *Code of Health and Disability Services Consumer’s Rights* (Health and Disability Commissioner 1996). The code outlines (among others) the right to freedom from coercion, the right to effective communication, the right to be fully informed, and the right to make an informed choice and give informed consent.

A discussion of the benefits and harms of suicide risk assessment is an important part of informed choice. Proposed benefits are speculative: they *may* prevent suicide or identify suicidal ideation. The utility of the test raises significant ethical issues about harm, however. The stratification to a high-risk group has the potential to cause significant anxiety for the person and their family. With a positive predictive value possibly as low as five per cent, it is questionable whether there are any health improvements to be had for most of this group to counter any psychological harm, particularly when we consider the absence of evidence for effective interventions. There is also the troubling matter that up to eighty-six per cent of people who die by suicide are stratified to low-risk groups (Appleby et al. 2006). This has the potential for providing false reassurance for those tested as low.

The World Health Organization’s own guidelines on screening acknowledges the need for the value of a screening test to be determined before it is introduced into practice, with a quantitative determination of premature death that can be prevented by screening, so that the benefits can be compared to the costs, both financial and human, including anxiety, follow up investigations, and treatments. They further note that identification of untreatable conditions can cause anxiety and waste resources with no practical outcome (Strong et al. 2005). Suicide risk assessments to detect and predict suicide do not meet these World Health Organization guidelines.

## Discussion

Suicide has significant, widely felt, harmful effects. Australian research has considered the financial costs of youth suicide, estimating that it has an economic cost of $511 million per year (Kinchin and Doran 2018). Then there are the more tangible negative emotional and health impacts on family and friends (Agerbo 2005; Erlangsen et al. 2017; Farberow et al. 1992). The goal of reducing suicide rates is a laudable one. The current health policy of widely used suicide risk assessment to detect, predict, and prevent suicide is built on key premises: that suicide equates to mental illness, that the assessments are effective at predicting suicide, and that interventions are effective. The evidence, reviewed here, does not sufficiently support these premises.

I have argued that the widespread and normative use of suicide risk assessments in health settings, as part of a suicide prevention policy, is consistent with a screening programme. When scrutinized using screening programme criteria, including recognized ethical criteria (informed choice, confidentiality, balancing of benefits/harms, respect for autonomy) this policy appears to be highly problematic. It systemically undermines autonomy, and by even the most conservative estimates, a large majority of those stratified as high risk of suicide are false-positive. Furthermore, there is a real risk of harm: from the psychological harm of being rated as high risk with potentially no health improvement to harm from increased rates of coercive interventions as a result of widespread screening and subsequent increased identification of “high-risk” individuals. This continued approach of widespread and normative use of suicide risk assessments to stratify patients into suicide risk categories and attempt to predict the probability of a suicide attempt is unethical. As such I believe that it should cease to be a component of suicide prevention policies and routine clinical practice.

What should be done in regard to risk assessments for the individual who has presented with suicidal ideation or behaviour? The temporary restriction of liberty of someone who appears intent on suicide, justified by the social contract or a Millsian approach, is not particularly controversial, but the movement of suicide prevention of individuals firmly into the domain of healthcare, relying on legislation for the compulsory treatment of mental illness, raises significant bioethical questions. Clearly there is a high association between suicide and mental illness, but the risk, as we have seen with the medical model, is that the two are conflated and that suicidal behaviour is treated as a mental illness and assumed non-autonomous in cases where there is insufficient justification to do so. This conflation does have appeal, transforming suicide from a complex issue into one with a straightforward health solution: treatment of mental illness, coercively if necessary. The problem is that there is no significant evidence of effectiveness for this approach. Large ([et al.] 2017; 2018) has identified this from his work on suicide risk assessment and concluded that people who present with mental illness should not be subjected to suicide risk assessments, as these assessments are not useful. Instead, they should be given an individualized assessment of their circumstances to determine their needs. I agree with this and have shown here that there are significant ethical problems with their use, particularly if the result is a coercive intervention. Despite the best intentions behind the intervention, there is no compelling evidence for beneficence. There is evidence of harm, however, and there is a disproportionate impact on disadvantaged groups, which compounds existing injustices. They also pose a risk to the principle of respect for autonomy.

It seems likely, in the absence of clear acceptable alternatives, that society will remain committed to using a Millsian-type approach to suicide prevention, and there will continue to be occasions, in clinical practice, when a coercive intervention is unavoidable. I would argue that these occasions become evident to the clinician as a result of an individualized psychiatric assessment, not through the use of a standardized risk assessment tool.

Increasing attention is being given to reducing cognitive biases and errors in medical decision-making (Croskerry 2003; Whelehan, Conlon, and Ridgway 2020). A recent systematic review of cognitive biases associated with medical decision-making identified that the anchoring effect, and availability biases were strongly associated with diagnostic inaccuracy (Saposnik et al. 2016). The availability bias is the tendency to use information that comes to mind quickly and easily (Tversky and Kahneman 1973). The anchoring effect is our tendency to weight too heavily the first piece of information we are given about a topic (Tversky and Kahneman 1974). The relevance here is in how the use of risk assessment tools, despite their lack of utility, can potentially influence clinical decision-making by introducing such biases and cognitive errors at the expense of statistical and logical judgement. The reality of clinical practice is that clinicians regularly face uncertainty when considering the appropriateness of temporarily restricting an individual’s liberty. Rather than enhancing decision-making in cases of uncertainty, it is likely that risk prediction and stratification instead introduce additional risk of cognitive bias: availability bias as a result of the initial stratification and anchoring in any future interaction once the risk assessment has been completed. In cases where there is uncertainty, the rating of high risk can add unjustified weight to a clinician’s decision-making at the expense of other relevant factors, including that the majority of people stratified as high risk will not go on to die by suicide, that there is a significant risk of harm from coercive intervention, and that the least restrictive approach is a fundamental principle in psychiatric practice (Royal Australian and New Zealand College of Psychiatrists 2017; New Zealand Ministry of Health 2020b). There appears to be a case that ceasing to use these risk assessments on individuals would reduce risk of cognitive errors that could promote decisions that result in ethically problematic and potentially inappropriate coercive intervention.

## Conclusion

The World Health Organization has stated that “every single life lost to suicide is one too many” (World Health Organization 2014) and it is “imperative” to address suicide (World Health Organization 2018). In a number of countries, including New Zealand and Australia, recent government inquiries have identified an urgent need to reduce suicide rates (Mental Health and Addiction Inquiry 2018; House of Representatives Select Committee on Mental Health and Suicide Prevention, 2021). In this context there has been an expansion in zero-suicide policies and, in healthcare, the use of suicide risk assessments.

I have shown here that there are significant problems in both systemic and individually used standardized suicide risk assessments, as a result of their utility and the ethics of their use. I believe that there is a strong case that they should cease to be used in clinical practice.

A zero-suicide policy is an understandable response to the “imperative” to reduce suicide. Unfortunately, there are no grounds to suggest that this is achievable. A single suicide may be preventable, but the idea that suicide can be eliminated as a phenomenon in society, which has to be the premise for zero-suicide policy, appears to be baseless. Although “zero suicide” has been defended as being “aspirational” (Mokkenstorm et al. 2018), this is not necessarily obvious, and intuitively a “zero suicide” strategy implies that it is in fact an achievable objective. The continued pursuit of this policy in health settings raises ethical questions of veracity, an important principle in its own right for some bioethicists (Veatch 2020).

It is also only a small step from the premises that underpin zero-suicide, to the expectation that suicidality is “treatable,” and suicide is therefore a failure of detection or treatment. Where “suicide prevention” is policy, there is frequent reference to the importance of training, resources, and skills to enable clinicians to improve their performance. One interpretation of this is that there is already such an expectation. Clinicians are sensitive to this, and it will affect their decision-making. Undrill (2011) describes the potentially harmful phenomenon that can occur of secondary risk assessment—essentially “clinician reputation management”— in response to concern about blame if a suicide were to occur. Refraining from attempting to predict risk could reduce the likelihood of this.

By being honest about the limitations of suicide prediction and prevention in mental health settings, there is the potential for improvement both in mental health care and suicide prevention. Limited mental health resources could be redirected away from attempting to implement zero-suicide or similar policies and towards improving access and treatment for people with mental illness. If access is improved, those people with mental illness who have, or are at risk of, suicidal thoughts would have increased opportunity for treatment. Healthcare may actually improve if the pressure for clinicians to predict risk is lowered.

A shift away from this approach would also allow for a greater focus on measures that do have demonstrable effects on suicide rates. Public health initiatives have been shown to influence suicide rates in a way that individual-targeted healthcare strategies have not.^4^ Similarly, community-based interventions targeting social isolation have been shown to reduce suicide rates (e.g., Motohashi et al. 2007). These measures are not cost-neutral and can be paternalistic, albeit at a societal rather than an individual level. They require political capital, though, and when governments go through cycles of austerity, social measures such as social clubs, community support services, public transport links, etc., are often among the first targets of cost-saving measures.

Finally, notwithstanding the “imperative” to prevent suicide and accompanying political and social pressures, when we consider the global trend in suicide, it is important to note that rates have considerably fallen, by thirty-six per cent over the last twenty years (World Health Organization 2021). This fact provides support to an argument that while the reduction of suicide is an important goal for society, there is no requisite to do so in disregard of the generally accepted medical principles of using evidence-based interventions, which have been subject to ethical approval.

## Footnotes

1 Health minister David Clark in his introduction to *Every Life Matters—He tapu te oranga o ia tangata: Suicide Prevention Strategy 2019–2029 and Suicide Prevention Action Plan 2019–2024 for Aotearoa New Zealand* (New Zealand Ministry of Health 2019)

2 A concept originating from humoral theory, the meaning of melancholia has evolved over time, with the pre–twentieth century meaning having little in common with current psychiatric usage. In nineteenth-century Britain this was a term initially wedded to the idea of a disorder of intellect where certain views were held with more prominence than normal, often, but not always associated with sadness. Over the course of a few decades there was a shift in view away from this and towards a view that melancholia was a disorder of mood (Berrios 1988; Kendler 2020).

3 “Positive predictive value” is the percentage of people who test positive who are positive. In the case of suicide, it would represent the proportion of people who test positive in a suicide risk assessment who go on to die by suicide. Positive predictive value (PPV) is influenced by the prevalence of a disease. The prevalence of suicide in a population is very low which will mean positive predictive value will be low. This is of relevance here as the studies described here are of populations of patients already under psychiatric care, populations felt to be at higher risk of suicide than the general population. If the tests were applied to the general population the PPV values would be infinitesimally small.

4 There has been convincing evidence of public health interventions reducing suicides by specific methods. Restrictions in access to handguns have been associated with reduced suicide by handgun and a drop in overall suicide rates (Anestis and Anestis 2015). In the United Kingdom a switch from using coal to natural gas reduced rates of suicide by carbon monoxide poisoning (Klonsky, May, and Saffer 2016) and restrictions around the quantity of paracetamol available for purchase corresponded with a decrease in deaths involving paracetamol overdose (Hawton et al. 2013).
